# Adjusting Mortality for Loss to Follow-Up: Analysis of Five ART Programmes in Sub-Saharan Africa

**DOI:** 10.1371/journal.pone.0014149

**Published:** 2010-11-30

**Authors:** Martin W. G. Brinkhof, Ben D. Spycher, Constantin Yiannoutsos, Ralf Weigel, Robin Wood, Eugène Messou, Andrew Boulle, Matthias Egger, Jonathan A. C. Sterne, for the International epidemiological Database to Evaluate AIDS (IeDEA)

**Affiliations:** 1 Division of International and Environmental Health, Institute of Social and Preventive Medicine (ISPM), University of Bern, Bern, Switzerland; 2 Division of Biostatistics, Indiana University, Indianapolis, Indiana, United States of America; 3 Lighthouse Trust at Kamuzu Central Hospital and Ministry of Health, Lilongwe, Malawi; 4 Faculty of Health Sciences, Desmond Tutu HIV Centre, Institute for Infectious Disease and Molecular Medicine, University of Cape Town, Cape Town, South Africa; 5 Programme PAC-CI, Abidjan, Côte d'Ivoire; 6 School of Public Health and Family Medicine, University of Cape Town, Cape Town, South Africa; 7 Department of Social Medicine, University of Bristol, Bristol, United Kingdom; INSERM, France

## Abstract

**Background:**

Evaluation of antiretroviral treatment (ART) programmes in sub-Saharan Africa is difficult because many patients are lost to follow-up. Outcomes in these patients are generally unknown but studies tracing patients have shown mortality to be high. We adjusted programme-level mortality in the first year of antiretroviral treatment (ART) for excess mortality in patients lost to follow-up.

**Methods and Findings:**

Treatment-naïve patients starting combination ART in five programmes in Côte d'Ivoire, Kenya, Malawi and South Africa were eligible. Patients whose last visit was at least nine months before the closure of the database were considered lost to follow-up. We filled missing survival times in these patients by multiple imputation, using estimates of mortality from studies that traced patients lost to follow-up. Data were analyzed using Weibull models, adjusting for age, sex, ART regimen, CD4 cell count, clinical stage and treatment programme. A total of 15,915 HIV-infected patients (median CD4 cell count 110 cells/µL, median age 35 years, 68% female) were included; 1,001 (6.3%) were known to have died and 1,285 (14.3%) were lost to follow-up in the first year of ART. Crude estimates of mortality at one year ranged from 5.7% (95% CI 4.9–6.5%) to 10.9% (9.6–12.4%) across the five programmes. Estimated mortality hazard ratios comparing patients lost to follow-up with those remaining in care ranged from 6 to 23. Adjusted estimates based on these hazard ratios ranged from 10.2% (8.9–11.6%) to 16.9% (15.0–19.1%), with relative increases in mortality ranging from 27% to 73% across programmes.

**Conclusions:**

Naïve survival analysis ignoring excess mortality in patients lost to follow-up may greatly underestimate overall mortality, and bias ART programme evaluations. Adjusted mortality estimates can be obtained based on excess mortality rates in patients lost to follow-up.

## Introduction

Loss to follow-up (LTFU) is an important problem both for the care of individual patients and the evaluation of antiretroviral treatment (ART) programmes in low- and middle-income countries. In a systematic review of programmes from sub-Saharan Africa the percentage of patients lost to follow-up was estimated to be 19%, 24% and 31% at 6 months, 12 months and 24 months of treatment, respectively [1]. Other studies have documented an increase in LTFU in more recent years, during which the number of patients starting ART increased steeply [2], [3]. LTFU is thus becoming an increasing problem in these settings, as programmes grow and staff-to-patient ratios decrease [4].

LTFU is also a problem for estimating outcomes at the level of the ART programme: a meta-analysis of studies that traced patients lost to follow-up to ascertain their vital status showed that in sub-Saharan Africa 46% of those traced had died [5]. These deaths generally occurred within the first months of ART, and death rates in the first year of ART are therefore considerably higher in patients lost to follow-up than the 7% to 13% commonly reported for ART programmes in lower-income countries [2], [6], [7], [8]. Standard methods of survival analysis that censor follow-up time at the last visit will therefore underestimate overall, programme-level mortality [9].

We propose an approach based on multiple imputation [10] to conduct sensitivity analyses adjusting estimates of cumulative mortality during the first year of ART for the excess risk of death in those lost to follow-up. Our analyses are based on five large ART programmes in sub-Saharan Africa that experienced different levels of LTFU.

## Methods

### Data sources

The International epidemiological Database to Evaluate AIDS (IeDEA) is a network of HIV/AIDS treatment programmes in Africa, North and South America and Asia. The collaboration has been described in detail elsewhere [5], [11], [12]. For the present analysis we used data from five treatment programmes in sub-Saharan Africa: the Centre de Prise en Charge de Recherches et de Formation (CePReF) from Abidjan, Côte d'Ivoire [8] (West African IeDEA Region); the Academic Model for the Prevention and Treatment of HIV/AIDS (AMPATH) from Eldoret, Kenya [13]] (East African IeDEA Region); the Lighthouse clinic in Lilongwe, Malawi [14] (Southern African IeDEA Region); and the township programmes in Khayelitsha [15] and Gugulethu [16], both in Cape Town, South Africa (Southern African IeDEA Region).

### Inclusion criteria and definitions

All treatment-naïve patients starting ART with documented age, sex, CD4 count and clinical stage at the start of treatment (baseline) and with at least one day of follow-up were included. ART was defined as any combination of three or more antiretroviral drugs. Follow-up time was measured from the start of ART and censored at the earliest of the date of death, the date of the last follow-up visit, or 12 months after starting ART. Patients were considered lost to follow-up if their last visit preceded the closure date of the database by nine months or more and no death had been recorded by that time. Nine months was obtained by adding three months (within which patients could have returned) to the maximum interval of six months between scheduled visits. Calculations of LTFU rates were therefore based on patients who started ART at least nine months before the closure date of the database. The outcome of interest was mortality from all causes in the first year of ART. We used an intention-to-continue treatment approach, ignoring changes to treatment, treatment interruptions and terminations.

### Estimation of mortality in patients lost to follow-up

To account for the excess risk of death in patients lost to follow-up we estimated, for each ART programme, a constant mortality hazard ratio comparing patients lost to follow-up with those not lost to follow-up. Henceforth, we refer to these hazard ratios as HR_LTFU_. Estimates were based on a meta-regression analysis of studies tracing patients lost to follow-up in sub-Saharan Africa that found a negative relationship between the overall rate of LTFU and mortality in patients lost to follow-up: mortality at one year among patients lost to follow-up declined from around 60% to 20% as LTFU increased from 5% to 50% [5]. We used the regression equation to estimate the one-year mortality risk among patients lost to follow-up that is predicted by the rate of LTFU in that programme. We then identified the HR_LTFU_ for each programme that produced estimated cumulative mortality at one year consistent with the predicted mortality among patients lost to follow-up. In a second step we conducted sensitivity analyses including a range of assumed HR_LTFU_. We chose a range of HR_LTFU_ between 1 (no informative censoring) and 40. This range is justified by the very high mortality that has been observed in some ART programmes among patients lost to follow-up [5].

### Multiple imputation of survival times

We treated LTFU as a missing data problem, and used multiple imputation [10] to fill in the missing survival times in patients lost to follow-up and hence to obtain estimates of one-year mortality that were adjusted for LTFU. Imputation of missing survival times was based on a pattern-mixture modelling approach, in which we stratify subjects by their pattern of missing values, in our case LTFU status, and formulate distinct imputation models for each stratum [17]. For patients retained in care we fitted a proportional hazards Weibull model using the original right-censored data. The mode included baseline covariates age (16–29; 30–39; 40–49; ≥50 years), gender, type of ART regimen (non nucleoside reverse transcriptase inhibitor (NNRTI)-based; protease inhibitor (PI)-based: other or unknown), baseline CD4 count (<25; 25–49; 50–99; 100–199; ≥200 cells/µL), clinical stage of disease (less advanced = WHO stage 1 or 2; advanced = WHO stage 3 or 4), and indicators for the treatment programme. Imputation of survival times in patients lost to follow-up was based on the same model but assumed that the hazard of death was increased by factor HR_LTFU_. The multiple imputation procedure consisted of four steps:

1. Fit a Weibull survival model to the original censored survival data.
2. Specify a value of HR_LTFU_. Randomly sample the time from LTFU to death in each patient lost to follow-up, based on the model fitted in step 1, with the hazard of death increased by factor HR_LTFU_. Censor follow-up at one year, if imputed survival extends to beyond one year.
3. Repeat this procedure 10 times, to create 10 datasets including imputed survival times for patients lost to follow-up.
4. Estimate mortality at one year for each of the 10 datasets and combine estimates using Rubin's rules [18] to obtain overall estimates of mortality at one year adjusted for bias due to LTFU.

Further technical details of this approach are given in the Appendix S1.

### Analysis of heterogeneity between programmes

Differences in cumulative mortality between programmes will be partly due to differences in the distribution of patient characteristics at baseline. We therefore also estimated cumulative mortality for a “typical patient group” whose baseline characteristics correspond to the most frequent category for each characteristic, i.e. female, age 30 to 39 years, NNRTI-based regimen, CD4 100 to 199 cells/µL, and advanced stage of disease (WHO stage 3 or 4). Finally, we compared coefficients of variation to investigate whether adjusting for predicted mortality in patients lost to follow-up reduced between-programme heterogeneity. All analyses were done using Stata version 10.1 (Stata Corporation, College Station, Texas, USA).

## Results

The five treatment programmes provided data on 15,915 patients, of whom 10,773 (68%) were women. Median age was 35 years (inter-quartile range [IQR] 29–41 years) and the median CD4 cell count at the start of ART was 110 cells/µL (IQR 45–182) (Table 1). A total of 1,001 deaths (6.3% of patients) were recorded during 10,265 person-years of follow-up and 1,285 (14.3%) patients were lost to follow-up in the first year of ART. This percentage ranged from 5.7% to 28.9% across the five treatment programmes.

**Table 1:** Characteristics of patients enrolled in different treatment programmes.

Treatment programme	Country	No eligible patients	No (%) women	Median age (IQR)	Person-years of follow-up in first year	No (%) deaths in first year	No (% of eligible#) lost to follow-up in first year
CePReF	Côte d'Ivoire	2518	1846 (73)	35 (30–42)	2010	189 (7.5)	269 (12.6)
AMPATH	Kenya	5491	3716 (68)	36 (30–43)	2580	181 (3.3)	333 (22.0)
Lighthouse	Malawi	2754	1647 (60)	36 (30–43)	1550	204 (7.4)	439 (28.9)
Gugulethu	South Africa	1872	1272 (68)	33 (29–39)	1247	137 (7.3)	87 (8.3)
Khayelitsha	South Africa	3280	2292 (70)	32 (28–38)	2878	290 (8.8)	157 (5.7)
Overall		15915	10773 (68)	35 (29–41)	10265	1001 (6.3)	1285 (14.3)

IQR, interquartile range. CePReF, Centre de Prise en Charge de Recherches et de Formation. AMPATH, Academic Model for the Prevention and Treatment of HIV/AIDS.

# Excluding patients with unknown follow-up status (not known to have died and a last visit date in the first year, but with less than nine months of additional follow-up until the closure date of the cohort).

Table 2 compares baseline CD4 count and disease stage in patients who were not lost to follow-up, patients who were known to have died and patients who were lost to follow-up in the first year of ART. Patients who died had lower median CD4 counts and more advanced disease at baseline, compared to the other two groups, in each of the treatment programmes. In patients lost to follow-up, the median baseline CD4 count and prevalence of advanced disease were intermediate between the patients who died and those not lost to follow-up. These patterns were similar across treatment programmes, with the exception that patients lost to follow-up had somewhat higher baseline CD4 counts in Gugulethu and the prevalence of advanced disease was slightly lower in patients lost to follow-up in Gugulethu and Lighthouse, compared with patients not lost to follow-up.

**Table 2:** Clinical characteristics at baseline of patients known to survive or die in the first year of antitretroviral therapy and patients lost to follow-up in the first year of therapy.

	Median CD4 count (IQR; cells/µL)			Percent with advanced disease (95% CI*)		
Treatment programme	Not lost, not dead in first year	Not lost, dead in first year	Lost to follow-up in first year	Not lost, not dead in first year	Not lost, dead in first year	Lost to follow-up in first year
CePReF	142 (64–223)	52 (13–136)	108 (29–198)	80% (78–82)	96% (93–98)	83% (78–88)
AMPATH	118 (48–193)	52 (11–122)	73 (20–165)	55% (53–56)	86% (80–91)	65% (59–70)
Lighthouse	140 (67–223)	52 (18–124)	93 (38–177)	86% (84–88)	95% (91–97)	82% (78–86)
Gugulethu	107 (56–162)	53 (16–111)	115 (48–177)	79% (77–81)	95% (90–98)	78% (68–86)
Khayelitsha	93 (41–149)	37 (13–97)	64 (29–120)	89% (88–90)	98% (96–99)	92% (86–96)
Overall	116 (51–186)	48 (14–115)	90 (29–170)	74% (73–75)	94% (93–96)	79% (76–81)

IQR, interquartile range. CePReF, Centre de Prise en Charge de Recherches et de Formation. AMPATH, Academic Model for the Prevention and Treatment of HIV/AIDS.

*Binomial exact confidence interval.

The crude estimates of cumulative mortality at one year (based on the original data with censoring of follow-up time in patients lost to follow-up), were 8.6% (95% CI 7.5–9.9%) in CePReF, 5.7% (4.9–6.5%) in AMPATH, 10.9% (9.6–12.4%) in Lighthouse, 9.6% (8.2–11.2%) in Gugulethu, and 9.3% (8.4–10.4%) in Khayelitsha. As expected, estimates from imputation models were similar when the assumed HR_LTFU_ was 1 (and therefore the censoring of follow-up time non-informative): 8.6% (7.5–9.8%) for CePReF, 5.9% (5.1–6.9%) for AMPATH, 10.8% (9.4–12.3%) for Lighthouse, 9.1% (7.7–10.7%) for Gugulethu and 9.3% (8.3–10.4%) for Khayelitsha (Table 3). In the typical patient group, assuming HR_LTFU_ = 1, estimated one-year mortality varied between 4.2% (3.4–5.2%) and 7.3% (6.0–8.9%) in the different programmes.

**Table 3:** Cumulative mortality (95% CI) at one year after starting ART for HR_LTFU_  = 1 and for HR_LTFU_ corresponding to the predicted percentage mortality among patients lost to follow-up for each programme.

Treatment programme	Predicted mortality (%) among LTFU*	Value of HR_LTFU_ corresponding to predicted mortality in LTFU	Mortality in all patients (%)			Mortality (%) in typical patient group		
			When HR_LTFU_ = 1	Corresponding to predicted HR_LTFU_	Relative increase	When HR_LTFU_ = 1	Corresponding to predicted HR_LTFU_	Relative increase
CePReF	51%	18	8.6 (7.5–9.8)	13.8 (12.4–15.4)	60%	6.2 (5.1–7.6)	10.1 (8.2–12.3)	63%
AMPATH	42%	12	5.9 (5.1–6.9)	10.2 (8.9–11.6)	73%	4.2 (3.4–5.2)	7.0 (5.7–8.6)	67%
Lighthouse	35%	6	10.8 (9.4–12.3)	16.9 (15.0–19.1)	56%	7.3 (6.0–8.9)	10.8 (8.8–13.2)	48%
Gugulethu	56%	23	9.1 (7.7–10.7)	12.2 (10.5–14.3)	34%	6.4 (5.1–7.9)	8.6 (7.0–10.5)	34%
Khayelitsha	58%	20	9.3 (8.3–10.4)	11.8 (10.7–13.0)	27%	5.3 (4.4–6.4)	6.7 (5.6–8.0)	26%

CePReF, Centre de Prise en Charge de Recherches et de Formation. AMPATH, Academic Model for the Prevention and Treatment of HIV/AIDS.

Estimates for the typical patient group were from Weibull models while estimates for programme-specific mortality were from Kaplan-Meier methods. The typical patient group had baseline characteristics age 30 to 39; female; NNRTI-based regimen; CD4 count 100 to 199 cells/µL; advanced stage of disease (WHO stage 3 or stage 4).

*Predicted from a meta-regression analysis of the relationship between mortality in patients lost to follow-up and the programme LTFU rate [5].

The meta-regression analysis suggested that for each 10% increase in the programme LTFU rate, the odds of deaths among patients lost to follow-up was multiplied by 0.67 [5]. Table 3 shows the mortality among patients lost to follow-up for each programme as predicted from the rate of LTFU in that programme, and the values of HR_LTFU_ that correspond to the predicted mortality. The lowest predicted HR_LTFU_ were 6 and 12, for Lighthouse and AMPATH respectively: these were the programmes with the highest LTFU rates (Table 1). The predicted HR_LTFU_ for the remaining three programmes, which had substantially lower rates of LTFU, ranged between 18 and 23. For the HR_LTFU_ corresponding to the predicted mortality in patients lost to follow-up, the adjusted mortality in all patients ranged from 10.2% (95% CI 8.9–11.6%) in AMPATH to 16.9% (15.0–19.1%) in Lighthouse. The corresponding range for adjusted mortality in the typical patient group was 6.7% (5.6–8.0%) to 10.8% (8.8–13.2%). The relative increase in mortality, compared to mortality when HR_LTFU_ = 1, varied from 27% to 73% overall, and from 26% to 67% in the typical patient group. The highest relative increases in mortality were observed for Lighthouse and AMPATH; the programmes with the highest rate of LTFU (Table 1).

**Figure 1:**
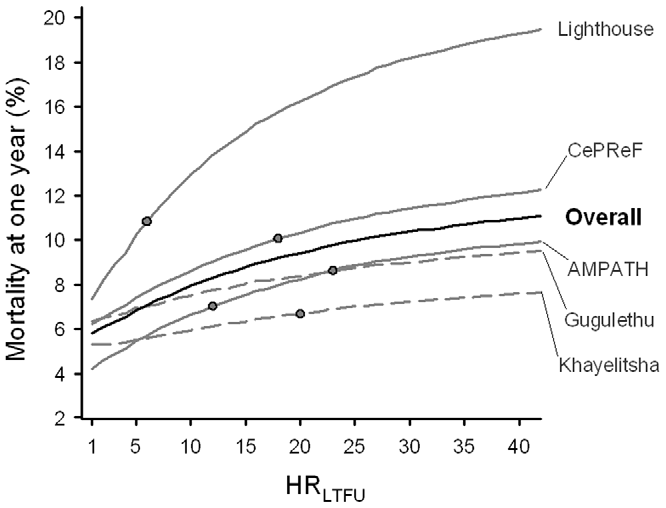
Relationship between assumed HR_LTFU_ and estimated mortality following outcome imputation in a typical patient group. The circles indicate the expected mortality in the typical patient group for the value of HR_LTFU_ corresponding to predicted mortality in patients lost to follow-up. CePReF, Centre de Prise en Charge de Recherches et de Formation. AMPATH, Academic Model for the Prevention and Treatment of HIV/AIDS.

Figure 1 shows the increases in overall and programme-specific estimated mortality at one year with increasing HR_LTFU_ in the typical patient group. For example, as the assumed HR_LTFU_ increased, mortality in the AMPATH programme changed from the lowest mortality to being close to the average estimate across programmes. In contrast, mortality in Lighthouse was higher than in the other programmes, regardless of the assumed HR_LTFU_. Variation in slopes between treatment programmes reflects differences in the proportion of patients lost to follow-up, the observed follow-up time for patients lost to follow-up, as well variation in covariates between treatment programmes. The shape of the curve reflects the use of proportional hazards in the Weibull models (see Appendix S1).

Table S1 gives estimates of mortality at one year with 95% CI in the typical patient group for different HR_LTFU_ separately for each programme and overall. When assuming that mortality rates in patients lost to follow-up were twice those in patients not lost to follow-up (HR_LTFU_  = 2), overall mortality in the typical patient group increased from 5.8% to 6.1% (5.2%–7.2%). Overall mortality further increased to 7.9% (6.7%–9.4%), 9.4% (8.0%–11.1%) and 11.0% (9.7%–12.4%) for HR_LTFU_ of 10, 20 and 40, respectively. Finally, adjusting for predicted mortality in patients lost to follow-up did not explain the between-programme variability in mortality: for the typical patient group, the coefficients of variation were 0.18 before adjustment for mortality in patients lost to follow-up and 0.19 after adjustment. The coefficients of variation for all patients were 0.18 and 0.17, respectively.

## Discussion

Mortality among patients lost to follow-up in ART treatment programmes in sub-Saharan Africa is high so that deaths reported for patients who remain in care may seriously underestimate mortality among all patients starting ART in a given programme [5]. By formulating this problem in terms of missing data we obtained adjusted mortality estimates, based on assumed hazard ratios for excess mortality in patients lost to follow-up. These sensitivity analyses are useful to estimate mortality among all patients starting ART, and to adjust prognostic models for bias due to informative censoring. Based on plausible estimates for excess mortality in patients lost to follow-up, programme-level mortality was increased by 27% to 73% overall, and 26% to 67% in patients with typical characteristics at the start of ART, with greater increases in programmes with higher rates of LTFU. Differences in rates of LTFU did not, however, explain variability in programme-specific mortality, even after accounting for patient characteristics at the start of ART.

Several ART programmes have recently traced patients lost to follow-up and used information on their vital status to derive adjusted mortality estimates. For example, in a cohort study of 410 patients in Gaborone (Botswana), the vital status of 46 (67.6%) of 68 patients lost to follow-up could be ascertained. Mortality increased from 7.1% before to 16.8% after tracing patients [19]. Geng and colleagues [20] traced a sample of 128 patients out of 829 patients lost to follow-up in Mbarara (Uganda), and obtained the vital status of 111 (87%) patients. Assuming that the latter were representative for all patients lost to follow-up, the authors used weighted Kaplan-Meier curves to obtain adjusted estimates: one-year mortality was 7.5%, compared to 1.7% before adjustment. Yiannoutsos and colleagues traced 1143 out of 3528 patients lost to follow-up in the Academic Model for the Prevention and Treatment of HIV/AIDS (AMPATH) programme in Eldoret (Kenya), and ascertained the vital status of 522 (54%) of those traced [21]. Using a double-sampling approach [22] the adjusted mortality estimate at one year was 10.7%, a six-fold increase compared to the unadjusted estimate [21].

The AMPATH programme was also included in our analysis: we found a mortality estimate at one year of 10.2%, similar to the double-sampling study [21], when we used the estimate for excess mortality in patients lost to follow-up from the meta-regression model [5]. In comparison the crude estimate (based on the original data with censoring of follow-up in patients lost to follow-up) for AMPATH was 5.7%. The double-sampling study in AMPATH thus validates our approach, indicating that in this programme the mortality rate in patients lost to follow-up is about 12 times greater than in patients not lost to follow-up (HR_LTFU_ = 12).

Several mechanisms might contribute to the higher mortality in patients lost to follow-up. First, patients might not return to the clinic because they have died. This is supported by the fact that patients with less favourable risk factor profiles at baseline (worse prognosis) are more likely to be lost to follow-up. Other possible reasons include incomplete adherence to ART [23], economic difficulties related to costs of transport and care [3], [24], [25], and HIV-related stigma [9], [24], [26]. The interruption or discontinuation of ART might then have led to disease progression and death. The limited evidence that is available indicates, however, that most deaths occurred shortly after the last clinic visit [5], [27], and are therefore probably related to opportunistic infections present at baseline. Deaths soon after starting ART are therefore likely to explain a large proportion of the excess mortality in patients lost to follow-up [28], [29]. In industrialized settings LTFU may have opposite effects if patients who feel well are more likely to leave the study than sick patients. For example, a French study found that patients with higher CD4 cell counts at baseline were more likely to be lost to follow-up [30]. Patients who returned to care after LTFU, however, experienced higher mortality than patients who attended clinics regularly [31].

Our approach has several strengths and limitations. It provides programme-specific estimates of mortality that are adjusted for differences in mortality rates between patients lost to follow-up and patients not lost to follow-up. Adjusted mortality can be computed for the whole population or for a particular covariate reference group. The latter facilitates comparison of adjusted mortality across different programmes, and avoids problems that occur when adjusting survival curves for confounders [32]. In contrast to linear regression, the use of centred covariates in survival analysis does not produce an estimate of average survival [32]. For this reason, we reported estimates of cumulative mortality for a group of patients with typical covariate values, adjusted for a range of assumed LTFU hazard ratios.

Deaths among patients lost to follow-up can be ascertained by tracing patients not returning to the clinic. Bias may thus be reduced or even abolished, but vital status often remains unknown in a substantial proportion of patients lost to follow-up, despite considerable efforts to trace them, and patients traced may therefore not be representative of all patients lost to follow-up [5]. Sensitivity analyses assuming different excess mortality ratios are useful in this situation, allowing the estimation of programme-level mortality for a range of plausible ratios.

With a cut-off of nine months our definition of LTFU was conservative and minimized the number of patients incorrectly classified as LTFU, i.e. the number of false positives. We will, however, have misclassified some patients as still being in care who were in fact lost to follow-up. A less conservative cut-off of six months would have increased the overall percentage of patients LTFU from 14.3% to 17.7%. Chi and colleagues recently examined the sensitivity and specificity of different definitions of LTFU in a large ART programme in Lusaka, Zambia [33]. They categorized LTFU on the basis of the number of days late for a scheduled visit, and determined the proportion of persons who returned to care within the subsequent year. Chi and colleagues found that for a cut-off of greater than 6 months, the sensitivity was 65.8% and the specificity 99.0% [33].

A limitation of our study is that we were not able to distinguish between LTFU and transfer of patients to another ART clinic: transfers out were not consistently recorded. Also, patients may self-transfer to another clinic without notifying the clinic where ART was initiated. Studies that traced patients lost to follow-up and documented reasons for LTFU indicate that such ‘silent’ transfers had occurred in 8% [25], 9% [24], 17% [34] and 19% [35] of patients. Transfer out may or may not be associated with increased or reduced mortality. For example if patients transfer entirely for practical reasons, for example to a clinic closer to their homes, then increased or reduced mortality is unlikely. Conversely, if patients are transferred for clinical reasons, for example to a higher or lower level of care, mortality is likely to differ. We now record transfers out and reasons for transfers systematically in IeDEA, allowing the imputation of survival times separately for patients lost to follow-up and patients transferred out in future analyses.

In conclusion, patients lost to follow-up in ART programmes in sub-Saharan Africa are at a substantially higher risk of death than patients who remain in care. LTFU in treatment programmes in sub-Saharan Africa is often substantial [1], [3] and therefore standard methods of survival analysis that censor patients at the time they are lost to follow-up may greatly underestimate overall mortality and bias programme evaluations. Therefore, sensitivity analyses adjusting mortality rates for plausible rates of excess mortality among patients lost to follow-up should be used in programme evaluations and in prognostic models. Future research should focus on ways to reduce LTFU, as well as on identifying factors responsible for the high risk of death in patients who do not remain in care, including undiagnosed opportunistic infections and cancers. A better understanding of these factors will contribute both to improve patient care and programme evaluation.

## Supporting Information

Table S1 Cumulative percentage of mortality (95% CI) in a typical patient group at one year after start of ART, for different assumed hazard ratios for mortality in patients lost to follow-up compared to patients not lost to follow-up (HR_LTFU_).
(0.04 MB DOC)

Appendix S1 Statistical appendix.
(0.11 MB DOC)
