# Supplementary material for: Adjusting Mortality for Loss to Follow-Up: Analysis of Five ART Programmes in Sub-Saharan Africa
Source: PLoS One. 2010 Nov 30;5(11):e14149. doi: 10.1371/journal.pone.0014149 (PMC2994756; doi:10.1371/journal.pone.0014149)
Supplement: Table S1 — Cumulative percentage of mortality (95% CI) in a typical patient group at one year after start of ART, for different assumed hazard ratios for mortality in patients lost to follow-up compared to patients not lost to follow-up (HRLTFU). (0.04 MB DOC) [file pone.0014149.s001.doc]

**Table S1**

|  | Treatment programme | | | | | | | | |  |  |
| --- | --- | --- | --- | --- | --- | --- | --- | --- | --- | --- | --- |
| HR_LTFU_ | CePReF |  | AMPATH |  | Lighthouse |  | Gugulethu |  | Khayelitsha |  | Overall |
| 1 | 6.2 (5.1-7.6) |  | 4.2 (3.4-5.2) |  | 7.3 (6.0-8.9) |  | 6.4 (5.1-7.9) |  | 5.3 (4.4-6.4) |  | 5.8 (5.0-6.8) |
| 2 | 6.5 (5.3-8.0) |  | 4.6 (3.7-5.7) |  | 8.2 (6.7-10.0) |  | 6.5 (5.2-8.0) |  | 5.3 (4.4-6.4) |  | 6.1 (5.2-7.1) |
| 4 | 7.1 (5.8-8.7) |  | 5.1 (4.1-6.3) |  | 9.5 (7.7-11.6) |  | 6.7 (5.4-8.4) |  | 5.4 (4.4-6.5) |  | 6.5 (5.5-7.7) |
| 6 | 7.7 (6.3-9.3) |  | 5.7 (4.7-7.0) |  | 10.8 (8.8-13.2) |  | 7.0 (5.6-8.7) |  | 5.6 (4.6-6.7) |  | 7.1 (6.0-8.3) |
| 8 | 8.2 (6.6-10.0) |  | 6.2 (5.0-7.7) |  | 11.9 (9.6-14.7) |  | 7.3 (5.8-9.1) |  | 5.8 (4.7-7.0) |  | 7.5 (6.3-9.0) |
| 10 | 8.6 (7.1-10.5) |  | 6.6 (5.4-8.2) |  | 13.0 (10.6-15.9) |  | 7.5 (6.0-9.4) |  | 6.0 (4.9-7.2) |  | 7.9 (6.7-9.4) |
| 15 | 9.5 (7.8-11.6) |  | 7.5 (6.2-9.2) |  | 14.9 (12.3-17.9) |  | 8.0 (6.5-9.9) |  | 6.3 (5.2-7.7) |  | 8.8 (7.4-10.4) |
| 20 | 10.3 (8.5-12.5) |  | 8.2 (6.8-10.0) |  | 16.2 (13.6-19.4) |  | 8.4 (6.8-10.3) |  | 6.7 (5.6-8.0) |  | 9.4 (8.0-11.1) |
| 25 | 11.0 (9.2-13.1) |  | 8.9 (7.4-10.6) |  | 17.3 (14.7-20.4) |  | 8.7 (7.2-10.6) |  | 7.0 (5.9-8.3) |  | 10.0 (8.6-11.5) |
| 30 | 11.4 (9.7-13.5) |  | 9.2 (7.8-10.9) |  | 18.2 (15.6-21.1) |  | 9.0 (7.5-10.8) |  | 7.2 (6.2-8.5) |  | 10.4 (9.1-11.9) |
| 40 | 12.1 (10.4-14.1) |  | 9.8 (8.4-11.6) |  | 19.3 (16.8-22.2) |  | 9.5 (7.9-11.3) |  | 7.6 (6.5-8.8) |  | 11.0 (9.7-12.4) |

CI, confidence interval. CePReF, Centre de Prise en Charge de Recherches et de Formation. AMPATH, Academic Model for the Prevention and Treatment of HIV/AIDS.

Analysis based on a typical patient group with baseline characteristics: age 30 to 39; female; non nucleoside reverse transcriptase inhibitor (NNRTI)-based regimen; CD4 count 100 to 199 cells/μL; advanced stage of disease (WHO stage 3 or stage 4).
