# Supplementary material for: Adjusting Mortality for Loss to Follow-Up: Analysis of Five ART Programmes in Sub-Saharan Africa
Source: PLoS One. 2010 Nov 30;5(11):e14149. doi: 10.1371/journal.pone.0014149 (PMC2994756; doi:10.1371/journal.pone.0014149)
Supplement: Appendix S1 — Statistical appendix. (0.11 MB DOC) [file pone.0014149.s002.doc]

**Brinkhof et al.: Loss to Follow-up and Mortality in Antiretroviral Treatment Programs in sub-Saharan Africa: Sensitivity Analysis Using Outcome Imputation**

**Appendix S1**

Standard uses of multiple imputation are based on an assumption that data are missing at random (MAR). However, when data are missing not at random (MNAR), multiple imputation may not correct biases in parameter estimates caused by the missing data.^1^ Pattern-mixture models^2-4^ can be used to estimate parameters under a range of MNAR assumptions. Our paper used multiple imputation to implement pattern mixture models^5^ in order to obtain estimates of 1 year mortality under various scenarios of increased risk in patients lost to follow up.

Expressed in terms of survival time *T* after initiation of ART the parameter of interest was$\mu=1-S\left(1\right)=P\left(T\leq1\right)$ where$S\left(1\right)=P\left(T>1\right)$ is the survival function evaluated at one year, i.e. the probability of survival beyond 1 year. Let *E* be an indicator for the event of death in the first year ($T\leq1$). Estimation of *μ* would be straightforward if *E* were observed for all patients. However, in many patients, including those lost to follow-up, *E* was not observed and only a right censored portion of *T* is observed. If this censoring were not related to the probability of death, i.e. if it were non-informative, the Kaplan-Meier estimator could be used to obtain an estimate for$\mu$. However, because subsequent death rates are likely to be higher after loss to follow up (informative censoring), mortality among patients still under observation is not representative of the original study population, resulting in a biased Kaplan-Meier estimate.

We used a pattern mixture model with two components, one modeling the risk of death in patients not lost to follow-up (not LTFU) and the other modeling risk in patients lost to follow-up (LTFU). We assume LTFU to be a separate process with event time denoted by *L*. For the first component we assume a proportional hazards Weibull model with hazard function

$h_{0}\left(t|Z\right)=p\exp\left\{c+\beta^{T}Z\right\}t^{p-1}$ [1]

where *t* is survival time, *c* is the regression constant (log of baseline hazard), *β* a vector of log hazard ratios, *Z* a vector of baseline covariates, and *p* is a shape parameter. In patients LTFU the following hazard function is assumed

$h_{1}\left(t|L,Z\right)=\left\{\begin{matrix} h_{0}\left(t|Z\right)\text{ if }t\leq L \\ kh_{0}\left(t|Z\right)\text{ if }t>L \end{matrix}\right.$ [2]

where *k* represents the mortality hazard ratio comparing patients lost to follow-up with patients not-lost to follow up (‘HR_LTFU_’ in the main paper), i.e. the degree of informative censoring. The other parameters are as in equation [1]. When *k* = 1 there is no informative censoring due to loss to follow-up, and *h_0_* = *h_1_*. When *k* > 1 patients going LTFU have a *k*-fold increased hazard from that moment on. Before that moment the hazard is entirely determined by the other model parameters *c*, *β* and *p* and is the same for all patients irrespective of whether they later become LTFU or not. These parameters can therefore be estimated by fitting a proportional hazards Weibull model to the right-censored data from all patients. The parameter *k* cannot be estimated from observable data and must be specified: we set *k* to integer values in the range 1 to 40.

We used multiple imputation to obtain mortality at 1 year, *μ*, from the model presented in equations [1] and [2] according to the following procedure:

1. Fit a Weibull survival model to the censored survival data to estimate parameters *c*, *β* and *p*.
2. Specify a value of *k*. Create $m=10$ imputed data sets by random sampling of survival time for patients LTFU using equation [2] as imputation model (see next section).
3. Estimate overall survival after one year $S(1)$in each of these data sets using the Kaplan-Meier estimator.
4. Combine the point and variance estimates of $\log(-\log(S(1))$from the imputed datasets using Rubin’s rules (see Rubin^6^ pp. 76 and Marshall et al^7^ to obtain, after back-transformation, estimated mortality rates and 95% confidence intervals.

The survivor function corresponding to equations [1] and [2] is given by

$S\left(t|L,Z\right)=\left\{\begin{matrix} \exp\left\{-\lambda\left(Z\right)t^{p}\right\}\text{ } \text{if } t\leq L \\ \exp\left\{-\lambda\left(Z\right)L^{p}-k\lambda\left(Z\right)\left(t^{p}-L^{p}\right)\right\} \text{ if } t>L \end{matrix}\right.$ [3]

where $\lambda\left(Z\right)=\exp\left(c+\beta^{T}Z\right)$. To create an imputation data set, we first randomly drew parameters *c*, *β*, and *p* from the (approximated) sampling distribution of the maximum likelihood estimators of these parameters,, i.e. from a multivariate normal distribution with mean $\hat{\theta}$ and covariance matrix equal to the inverse of the Fisher information matrix. For each patient LTFU within a year after initiation of ART we sampled survival time *T* from the conditional distribution of *T* given patient’s survival up to censoring time *L* and given the drawn parameters:

$F\left(t|t>L,Z\right)=1-\frac{S\left(t|L,Z\right)}{S\left(L|L,Z\right)}=1-\exp\left\{-k\lambda\left(Z\right)\left(t^{p}-L^{p}\right)\right\}$ [4]

To sample from this distribution we first drew a random deviate *U* from the uniform distribution on the interval $\left[0,1\right]$ and obtained a deviate *T* from the inverse of the above distribution function:

$$T=F^{-1}\left(U|t>L,Z\right)=\left\{L^{p}-\frac{1}{k\lambda\left(Z\right)}\ln\left(1-U\right)\right\}^{\frac{1}{p}}$$

The sampled survival times in patients LTFU and the observed survival times in patients not LTFU formed the imputation data set. Combining one year survival from the imputed data sets corresponds to averaging out S $S\left(1|L,Z\right)$ over LTFU status, LTFU times, *L*, and covariates, *Z*, to obtain estimates marginal one year survival$S\left(1\right)$ and consequently of one year mortality *μ*.

From equation [4] one can obtain a simple relationship between the probabilities of survival at 1 year in patients going LTFU (within the first year), $S_{1}\left(1|L,Z\right)=S\left(1|L<1,Z\right)$, and patients not LTFU, $S_{0}\left(1|Z\right)=S\left(1|L>1,Z\right)$:

$$S_{1}\left(1|L,Z\right)=\exp\left\{-\left(k-1\right)\lambda\left(Z\right)\left(1-L^{p}\right)\right\}S_{0}\left(1|Z\right)$$

Consequently one year conditional mortality in patients LTFU (conditional on covariates and LTFU time), $\mu_{1}\left(L,Z\right)$, can be written as a function of conditional mortality in patients not LTFU, $\mu_{0}\left(Z\right)$:

$\mu_{1}\left(L,Z\right)=1-S_{1}\left(1|L,Z\right)=1-\exp\left\{-\left(k-1\right)\lambda\left(Z\right)\left(1-L^{p}\right)\right\}\left(1-\mu_{0}\left(Z\right)\right)$. [5]

Averaged over *L*, the relationship between$\mu_{1}\left(Z\right)$and$\mu_{0}\left(Z\right)$ will deviate from this. Nevertheless, equation [5] governs the overall shape of the curves in Figure 1 which shows estimated overall mortality, $\mu\left(Z\right)=\left(1-r\right)\mu_{0}\left(Z\right)+r\mu_{1}\left(Z\right)$ (*r* represents the proportion of patients lost to follow-up) as a function of *k* for a common value of *Z*. For further details see Egger et al^8^ and Fenner et al.^9^

Reference List

(1) Sterne JA, White IR, Carlin JB, Spratt M, Royston P, Kenward MG et al. Multiple imputation for missing data in epidemiological and clinical research: potential and pitfalls. BMJ 2009; 338:b2393.

(2) Little RJA. Pattern-Mixture Models for Multivariate Incomplete Data. J Am Stat Assoc 1993; 88(421):125-134.

(3) Rubin DB. Inference and missing data. Biometrika 1974; 63:581-592.

(4) Daniels MJ, Hogan JW. Missing data in longitudinal studies: strategies for Bayesian modeling and sensitivity analysis. New York: Chapman & Hall/CRC; 2008.

(5) Demirtas H, Schafer JL. On the performance of random-coefficient pattern-mixture models for non-ignorable drop-out. Stat Med 2003; 22(16):2553-2575.

(6) Rubin DB. Multiple Imputation for Nonresponse in Surveys. New York: John Wiley; 1987.

(7) Marshall A, Altman DG, Holder RL, Royston P. Combining estimates of interest in prognostic modelling studies after multiple imputation: current practice and guidelines. BMC Med Res Methodol 2009; 9:57.

(8) Egger M, Spycher B, Sidle J, Messou E, Van Cutsem G, Weigel G et al. Correcting mortality for loss to follow up: a graphical approach applied to ART programmes in resource-limited settings. 5th IAS Conference on HIV Pathogenesis, Treatment and Prevention Cape Town, July 19-22, 2009 2009; Abstract WEPED173.

(9) Fenner L, Brinkhof MW, Keiser O, Weigel R, Cornell M, Moultrie H et al. Early Mortality and Loss to Follow-up in HIV-Infected Children Starting Antiretroviral Therapy in Southern Africa. J Acquir Immune Defic Syndr 2010;54(5):524-32.
